# Generation of a highly specific and potent antibody against Ce-lamin/LMN-1

**DOI:** 10.17912/micropub.biology.001294

**Published:** 2024-08-15

**Authors:** Adam Zahand, Byron Williams, Jun Liu

**Affiliations:** 1 Department of Molecular Biology and Genetics, Cornell University, Ithaca, NY, USA

## Abstract

High quality antibodies are useful tools for research in cell and developmental biology. We have obtained highly specific and potent guinea pig-derived polyclonal antibodies against Ce-lamin/ LMN-1 . Western blotting experiments using these antibodies demonstrated that maternally loaded Ce-lamin/ LMN-1 protein is very stable, can perdure through embryonic and larval development and remain detectable in homozygous null *lmn-1* mutant adults.

**Figure 1. Western blotting and immunofluorescence staining results showing the specificity and potency of anti-Ce-lamin (anti-LMN-1) antibodies from guinea pig CUMC-GP28.**
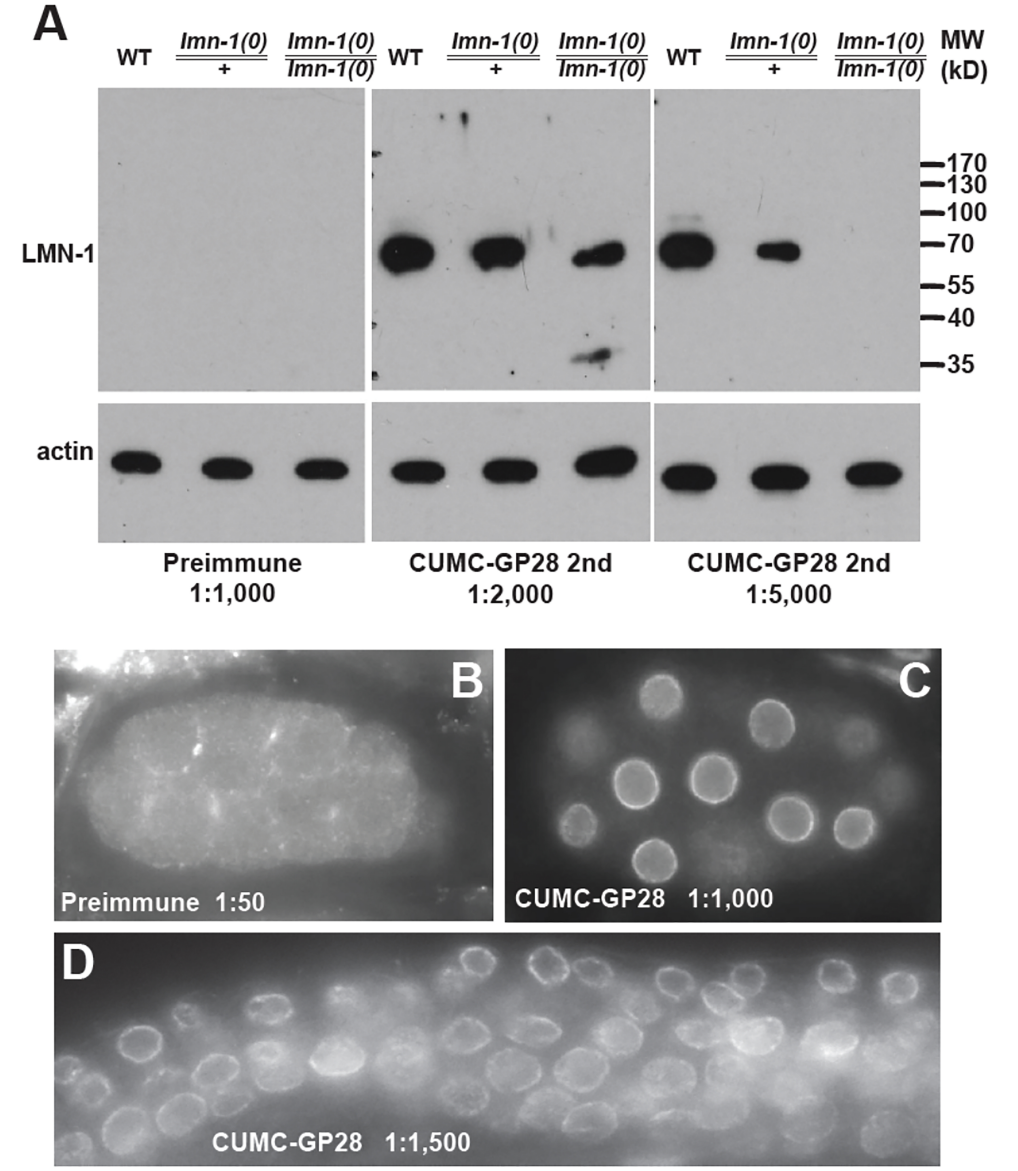
**A)** Sera from CUMC-GP 28 was tested against samples of wild-type ( N2 ), *lmn-1 (0)/+* ( *hT2 [ qIs48 ]/ lmn-1 ( tm1502 )),* and *lmn-1 (0)/ lmn-1 (0) ( lmn-1 ( tm1502 )/ lmn-1 ( tm1502 ))* adult animals. Preimmune serum was tested at 1:1,000 dilution, and second bleed serum was tested at 1:2,000 and 1:5,000. Arrow indicates Ce-lamin (with an approximate size of 70kD). **B-D** ) Immunofluorescence staining using preimmune (1:50, B), second bleed serum from CUMC-GP 28 (1:1,000, C) on N2 embryos, and second bleed serum from CUMC-GP 28 (1:1,500, D) on N2 L3 germline (see materials and methods). Image shown in panel C was taken at 0.5X exposure time used for the image shown in panel B.

## Description

The ability to generate animals expressing transgenic or endogenous, fluorescently-tagged proteins has made *C. elegans* a superb model for dissecting cell and developmental mechanisms in live animals at single cell resolution (Corsi et al. 2015). Nevertheless, high quality antibodies provide alternative tools for studying the distribution, biochemical properties, and functions of the corresponding proteins. These antibodies can also be used to mark specific subcellular structures or compartments in the developing animal.

The nuclear envelope is the defining boundary between the cytoplasm and the nucleoplasm of the cell. It consists of both inner and outer membranes, pore complexes for cross-membrane transport, and the nuclear lamina. The major component of the nuclear lamina is a meshwork of type V intermediate filament proteins called lamins. Lamins are critical for maintaining structural integrity of the nucleus, as well as proper genome organization and gene expression (de Leeuw et al. 2018). Mutations in the human LMNA gene can cause a wide variety of diseases that are collectively called laminopathies (Buxboim et al. 2023). *C. elegans* has a single gene encoding Ce-lamin/ LMN-1 (Riemer et al. 1993; Liu et al. 2000) . Knocking down of Ce-lamin/ LMN-1 results in severe abnormalities in nuclear morphology and the mis-localization of multiple nuclear envelope proteins (Lee et al. 2000; Liu et al. 2000; Gruenbaum et al. 2002; Lee et al. 2002; Liu et al. 2003; Haithcock et al. 2005; Margalit et al. 2005) . To facilitate further studies of Ce-lamin/ LMN-1 and provide another reagent to mark the nuclear envelope, we have generated highly specific and potent polyclonal antibodies against Ce-lamin.

As described in Methods, we immunized two guinea pigs with purified GST-Ce-lamin/ LMN-1 fusion proteins and tested the sera by both western blotting and immunofluorescence staining. Animal CUMC-GP28 generated highly specific and potent antibodies to Ce-lamin/ LMN-1 . As shown in Figure 1, preimmune serum from CUMC-GP28 detected no signal on western blot at 1:1,000 dilution ( Figure 1A ) and no nuclear envelope signal at 1:50 dilution via immunofluorescence staining ( Figure 1B ). In contrast, the second bleed from CUMC-GP28 specifically recognized a protein of predicted size for Ce-lamin/ LMN-1 at around 70 kD ( Figure 1A ), and showed strong nuclear envelope staining even at 1:1,000 dilution ( Figure 1C ), and 1:1,500 dilution ( Figure 1D ). Higher dilutions of CUMC-GP28 serum could possibly be used in immunofluorescence staining. However, the potency of higher dilutions was not tested. The serum from the final bleed showed strong signals on western blots too, even when tested at 1:20,000 dilution.

To further test for the specificity of the CUMC-GP28 serum, we performed western blotting using a previously described deletion allele of *lmn-1 , tm1502* (Haithcock et al. 2005) . *lmn-1 ( tm1502 )* homozygous animals are sterile and need to be maintained as a balanced heterozygous strain, *hT2 [ qIs48 ]/ lmn-1 ( tm1502 ),* which we denoted as *lmn-1 ( tm1502 )/+* . When lysates of the same number of stage-matched adult animals (as shown by the actin controls) were examined using western blotting, heterozygous *lmn-1 ( tm1502 )/+* animals had less Ce-lamin/ LMN-1 compared to wild-type animals ( Figure 1A ). With 1:5,000 dilution of the CUMC-GP28 serum, there was no detectable Ce-lamin/ LMN-1 in homozygous *lmn-1 ( tm1502 )/ lmn-1 ( tm1502 )* animals ( Figure 1A ), providing strong evidence demonstrating the specificity of the anti-Ce-lamin/ LMN-1 antibody. Surprisingly, a low level of Ce-lamin/ LMN-1 was still detectable in homozygous *lmn-1 ( tm1502 )/ lmn-1 ( tm1502 )* animals when the serum was used at 1:2,000 dilution, suggesting that homozygous *lmn-1 ( tm1502 )/ lmn-1 ( tm1502 )* adult animals still have residual amounts of Ce-lamin/ LMN-1 . This result suggests that Ce-lamin/ LMN-1 protein is very stable and perdures in homozygous adults from maternal load provided by *lmn-1 ( tm1502 )/+* heterozygous parents. This observation also explains why homozygous *lmn-1 (0)* null animals produced by heterozygous parents can survive through adulthood, albeit being sterile.

In summary, we have generated highly specific anti-Ce-lamin/ LMN-1 antibodies with high potency, without any need for pre-adsorption or further purification steps. Our western blotting results using these antibodies provide strong evidence demonstrating that maternally-loaded proteins, at least for Ce-lamin/ LMN-1 , can perdure through embryonic and larval development into adulthood in homozygous null animals.

## Methods

**Purification of GST::Ce-lamin**

pJKL409.3, which contains the open reading frame of Ce-lamin/ LMN-1 cloned into pGEX-2T via XmaI sites, was transformed into BL21. Four liters of log phase BL21 expressing pJKL409.3 was induced with 1 mM IPTG for ~4 hours, shaking at 37°C. Cells were harvested by centrifugation at 7,700 x g in a Sorvall centrifuge and resuspended in 100 ml of ice cold 1X PBS. Cells were portioned into four 50 ml tubes, and ¼ tablet of Complete, Mini Protease Inhibitor (Roche) was added to each bottle and allowed to dissolve. Each tube was sonicated on ice with a Markson Ultrasonic Processor with 6 bursts of 30 seconds each, at tune 40, with cooling time between each burst. 20% Triton X-100 was added to each bottle to 1% final concentration, and the bottles were allowed to rock gently for 30 minutes at room temperature. Bottles were centrifuged for 10 min at 4°C at 12,000 x g. Supernatant was saved to a new tube, and the pellet was resuspended in equal volume of 1X PBS.

Preparation of Glutathione Sepharose ^TM^ 4B beads and binding and elution of protein was carried out per manufacturer's instructions (Amersham Biosciences). Ethanol was removed from fresh bead slurry and beads were washed in 10X volume of ice cold 1X PBS three times and allowed to incubate in 1X PBS for ~2 hours, rocking at 4°C. Sonicated supernatant was added to prepared Glutathione Sepharose 4B beads and allowed to incubate while rocking gently at room temperature for 30 minutes. Beads were collected by centrifugation at 500 x g for 3 minutes and washed three times in 10X volume of 1X PBS. 1 ml of GST elution buffer (10 mM reduced glutathione in 50 mM Tris-HCl, pH 8.0) was added to the sample and was allowed to rock for 10 minutes at room temperature. The beads were sedimented by centrifugation at 500 x g for 3 minutes, and the eluate was collected to clean tubes. Elution was repeated twice more and eluates were pooled. A total of 6ml of purified protein was obtained and was subsequently concentrated by dialysis using polyethylene glycol and resuspended in 200 μl of 1X PBS.

**Antibody production**

A total of 700 μg of purified GST::Ce-lamin protein was provided to Cocalico Biologicals (Reamstown, PA) for antibody production in two guinea pigs, CUMC-GP 27 and CUMC-GP 28. A total of four boosts were given to each guinea pig before exsanguination.

**Storage of anti-Ce-lamin antibodies**

Upon receipt from Cocalico Biologicals (Reamstown, PA), antibodies against Ce-lamin were stored at -80°C. Preimmune, first bleed, and second bleed sera from both CUMC-GP 27 and CUMC-GP 28 were aliquoted into 100 μl portions and stored at -80°C, with 100 μl stored at -20°C in a 50% glycerol solution as a working stock. Third bleed serum from CUMC-GP 27 was aliquoted similarly, and sodium azide was added to the working stock to 0.02% final concentration. Final bleed serum from CUMC-GP 27 was left unaliquoted and stored at -80°C. Final bleed serum from CUMC-GP 28 was aliquoted into 500 μl portions and stored at -80°C. A 500 μl working stock in a 50% glycerol solution containing 0.02% sodium azide was stored at -20°C.

**Western blotting**

Age-synchronized N2 , *hT2 [ qIs48 ]/ lmn-1 ( tm1502 ),* and *lmn-1 ( tm1502 )/ lmn-1 ( tm1502 )* adult animals were collected (50 animals each) into 11 μl of 1X PBS, with an equal volume of 2X SDS sample buffer added. Proteins were transferred to Immobilin-P membranes (Millipore). Confirmation of GST::Ce-lamin fusion protein was performed by blotting with anti-GST antibody (Amersham Biosciences) at 1:2,000 dilution. Evaluation of bleeds from CUMC-GP 27 and CUMC-GP 28 was performed using varying dilutions of serum. Secondary antibodies used were HRP-conjugated anti-goat or HRP-conjugated anti-guinea pig IgG (Jackson ImmunoResearch). Both secondary antibodies were used at 1:10,000 dilution. As a loading control, blots were stripped using Restore Western Strip Buffer (Pierce) and re-probed with antibodies against actin. Mouse anti-actin (JLA20) was used in 1:2,000 dilution, and HRP-conjugated anti- mouse IgM was used as a secondary probe at 1:10,000. JLA20 was obtained from the Developmental Studies Hybridoma Bank, developed under the au­­spices of the National Institute of Child Health and Human Development and maintained by the University of Iowa Department of Biological Sciences (Iowa City).

**Immunofluorescence staining**

For testing of CUMC-GP 27 and CUMC-GP 28 sera, N2 , *hT2 [ qIs48 ]/ lmn-1 ( tm1502 ),* and *lmn-1 ( tm1502 )/ lmn-1 ( tm1502 )* animals were collected and fixed for indirect immunofluorescence microscopy as described (Liu *et al.* 2000). Serum was tested in varying dilutions. Images were visualized using a Leica DMRA2 compound microscope, with images captured using a Hamamatsu Orca-ER camera in conjunction with OPENLAB software (version 3.0.9, Improvision).
